# Investigation on the morphological and optical evolution of bimetallic Pd-Ag nanoparticles on sapphire (0001) by the systematic control of composition, annealing temperature and time

**DOI:** 10.1371/journal.pone.0189823

**Published:** 2017-12-18

**Authors:** Puran Pandey, Sundar Kunwar, Mao Sui, Sushil Bastola, Jihoon Lee

**Affiliations:** 1 College of Electronics and Information, Kwangwoon University, Nowon-gu, Seoul, South Korea; 2 Institute of Nanoscale Science and Engineering, University of Arkansas, Fayetteville, Arkansas, United States of America; Institute of Materials Science, GERMANY

## Abstract

Multi-metallic alloy nanoparticles (NPs) can offer additional opportunities for modifying the electronic, optical and catalytic properties by the control of composition, configuration and size of individual nanostructures that are consisted of more than single element. In this paper, the fabrication of bimetallic Pd-Ag NPs is systematically demonstrated via the solid state dewetting of bilayer thin films on c-plane sapphire by governing the temperature, time as well as composition. The composition of Pd-Ag bilayer remarkably affects the morphology of alloy nanostructures, in which the higher Ag composition, i.e. Pd_0.25_Ag_0.75_, leads to the enhanced dewetting of bilayers whereas the higher Pd composition (Pd_0.75_Ag_0.25_) hinders the dewetting. Depending on the annealing temperature, Pd-Ag alloy nanostructures evolve with a series of configurations, i.e. nucleation of voids, porous network, elongated nanoclusters and round alloy NPs. In addition, with the annealing time set, the gradual configuration transformation from the elongated to round alloy NPs as well as size reduction is demonstrated due to the enhanced diffusion and sublimation of Ag atoms. The evolution of various morphology of Pd-Ag nanostructures is described based on the surface diffusion and inter-diffusion of Pd and Ag adatoms along with the Ag sublimation, Rayleigh instability and energy minimization mechanism. The reflectance spectra of bimetallic Pd-Ag nanostructures exhibit various quadrupolar and dipolar resonance peaks, peak shifts and absorption dips owing to the surface plasmon resonance of nanostructures depending on the surface morphology. The intensity of reflectance spectra is gradually decreased along with the surface coverage and NP size evolution. The absorption dips are red-shifted towards the longer wavelength for the larger alloy NPs and vice-versa.

## Introduction

Metallic nanoparticles (NPs) play key roles in various applications such as solar cells, sensors and catalysis due to their plasmonic property, chemical stability, and larger surface to volume ratio. [1–6]. Bimetallic NPs with more than one element in an individual nanostructure can offer further opportunities for the enhanced performance due to their electronic heterogeneity, multi-functionality and specific site response [7–11]. Specially, the noble bimetallic NPs exhibits the improved optical absorption because of localized surface plasmon resonance (LSPR) resulting from the coherent oscillations of the conduction electrons within the NPs [12, 13]. For instance, a broader light absorption of dye molecules was exhibited by the Au-Ag alloy NPs due to the LSPR induced by both Au and Ag components, which resulted in the conversion efficiency improvement of dye-sensitized solar cells [13]. Also, significantly enhanced catalytic activity was demonstrated by the Au-Pd alloy NPs due to the electronic heterogeneity, which gave a rise to a stronger interaction of alloy NPs and reactant molecules [14]. Among various metallic elements, Pd and Ag alloy can find the potentials to improve the performance of optical, sensing and catalysis applications owing to the strong catalytic activity of Pd and plasmonic resonance of Ag as well as electronic heterogeneity of alloy NPs [15–18]. Such bimetallic alloy NPs can be fabricated by various chemical and physical growth technique [19–20]. Among them, solid stage dewetting of bilayer films can be an efficient method for the fabrication of bimetallic alloy nanostructures because of the high miscibility of Pd and Ag at every composition [21, 22]. Therefore, the systematic investigation on the morphological and optical properties of Pd-Ag alloy NPs on sapphire (0001) by the solid state dewetting can be of important groundwork, which, however, has not been reported yet. In this work, the evolution of various bimetallic Pd-Ag nanostructures is demonstrated on c-plane sapphire through the solid state dewetting of Pd-Ag bilayers. By the control of annealing temperature, time and composition, various configurations, size and density of composite Pd-Ag nanostructures are demonstrated and discussed based on the surface diffusion and inter-diffusion, Ag sublimation, Rayleigh instability, energy minimization mechanism and dewetting. The optical characterization of corresponding Pd-Ag nanostructures in term of reflectance spectra reveal the formation of various quadrupolar, dipolar peaks and absorption dips based on the surface plasmon resonance of bimetallic Pd-Ag nanostructures. The variation in the intensity and shift of reflectance peaks and absorption dips are disused accordingly with the surface morphology.

## Materials and methods

In this project, initially the 430 micron-thick sapphire (0001) wafers with an off-axis of ± 0.1° (iNexus Inc., South Korea) were diced in a square of 6 × 6 mm^2^ by a machine saw. Prior to the bilayer film deposition, the substrate was degassed at 700°C for 15 min under 1 × 10^−4^ Torr in a pulsed laser deposition (PLD) chamber to remove the surface contaminants such as particles, vapors, trapped air, and oxides. Surface morphology and optical characteristics of the bare sapphire after degassing are shown in S1 Fig. Then, the fabrication of bimetallic Pd-Ag nanostructures was carried out by the deposition of various composition of Pd and Ag films followed by the subsequent annealing. Three compositions of Pd-Ag films were prepared on sapphire with the total thickness of 6 and 20 nm, i.e. Pd_0.25_Ag_0.75_, Pd_0.5_Ag_0.5_ and Pd_0.75_Ag_0.25_. The Pd film was first deposited on sapphire followed by the Ag film at a growth rate of 0.1 nm/s with the 5 mA ionization current under the vacuum of 1 × 10^−1^ Torr. After the deposition, the samples were annealed in a PLD chamber at various conditions under the vacuum 1 × 10^−4^ Torr. To investigate the effect of annealing temperature on the evolution of bimetallic Pd-Ag nanostructures, there compositions of Pd-Ag bilayers (with total thickness 6 nm) samples were annealed between 400 and 900°C for 120 s by reaching the target temperature at the ramping rate of 4°C/s. For the study of annealing time behavior, samples with 20 nm thickness were annealed at 850°C for various annealing duration (Pd_0.25_Ag_0.75_, Pd_0.5_Ag_0.5_ and Pd_0.75_Ag_0.25_). The ramping rate was set to be 10°Cs in order to minimize the ripening. The surface morphology of bimetallic Pd-Ag nanostructures was characterized by an atomic force microscope (AFM) (XE-70, Park Systems Corp., South Korea) and scanning electron microscope (SEM) (CX-200, COXEM, South Korea). The AFM was operated under a non-contact mode at ambient with the same batch of AFM tips (NSC16/AIBS, MikroMasch, USA). The elemental analysis was performed by an energy-dispersive x-ray spectroscope (EDS) system under vacuum with the spectral and mapping modes (Noran System 7, Thermo Fisher, USA). 532 nm laser at 220 mW was utilized for Raman spectra and deuterium and halogen lamps were used for the UV-VIS-NIR reflectance spectra at ambient (UNIRAM II system, UniNanoTech Co. Ltd, South Korea).

## Results and discussion

**Fig 1:**
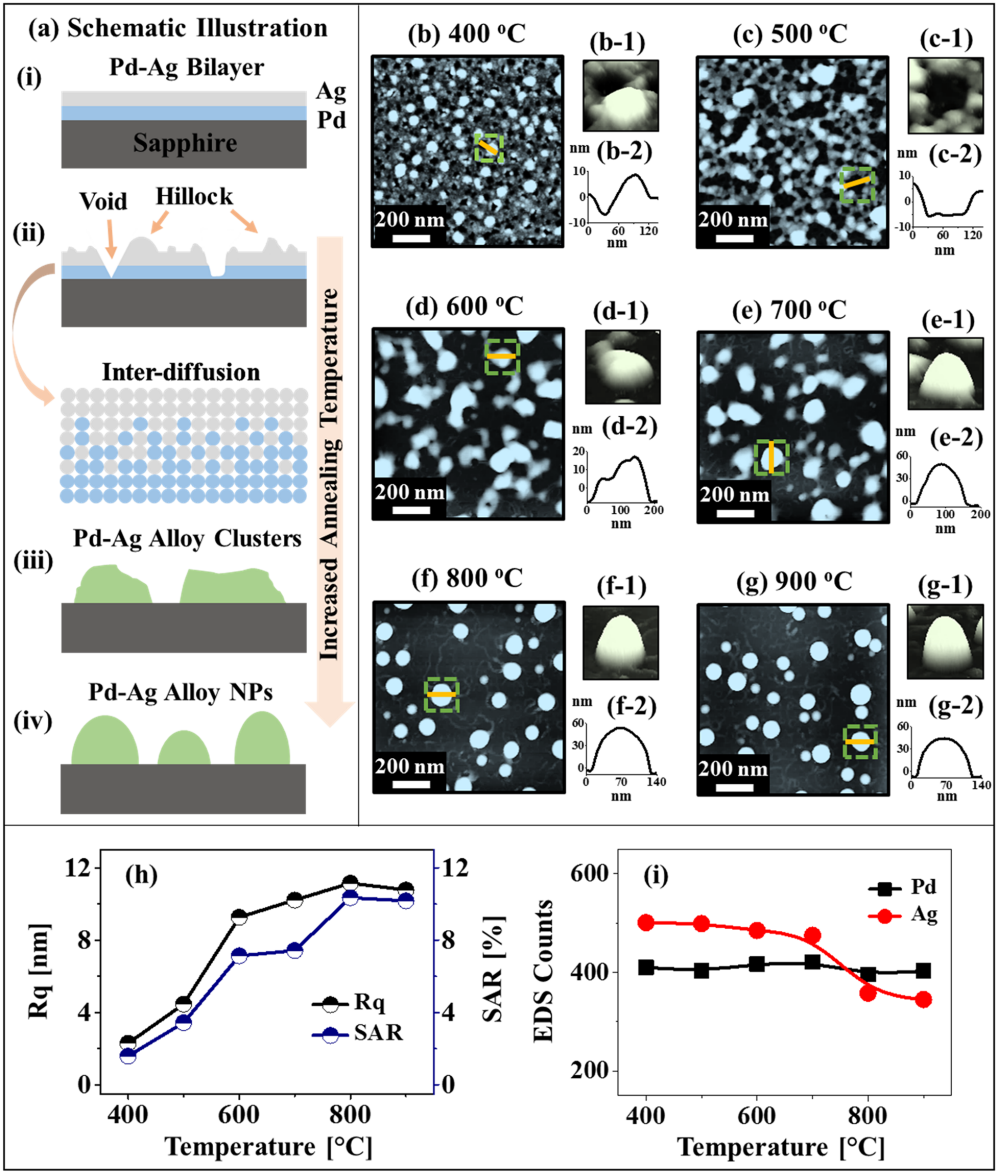
Evolution of various bimetallic Pd-Ag nanostructures by the control of annealing temperature between 400 and 900°C on sapphire (0001) with a fixed total thickness 6 nm, composition Pd_0.5_Ag_0.5_ and annealing time 120 s. (a) Schematic illustration showing the evolution process of Pd-Ag alloy NPs. (b)–(g) AFM top-views (1 × 1 μm^2^) of voids, nanoclusters and round dome shaped Pd-Ag alloy NPs. (b-1)–(g-1) AFM side-views (b-2)–(g-2) Cross-sectional line-profiles. (h) Plot of RMS surface roughness (Rq) and surface area ratio (SAR). (i) Plot of Ag Lα1 and Pd Lα1 EDS count.

**Fig 2:**
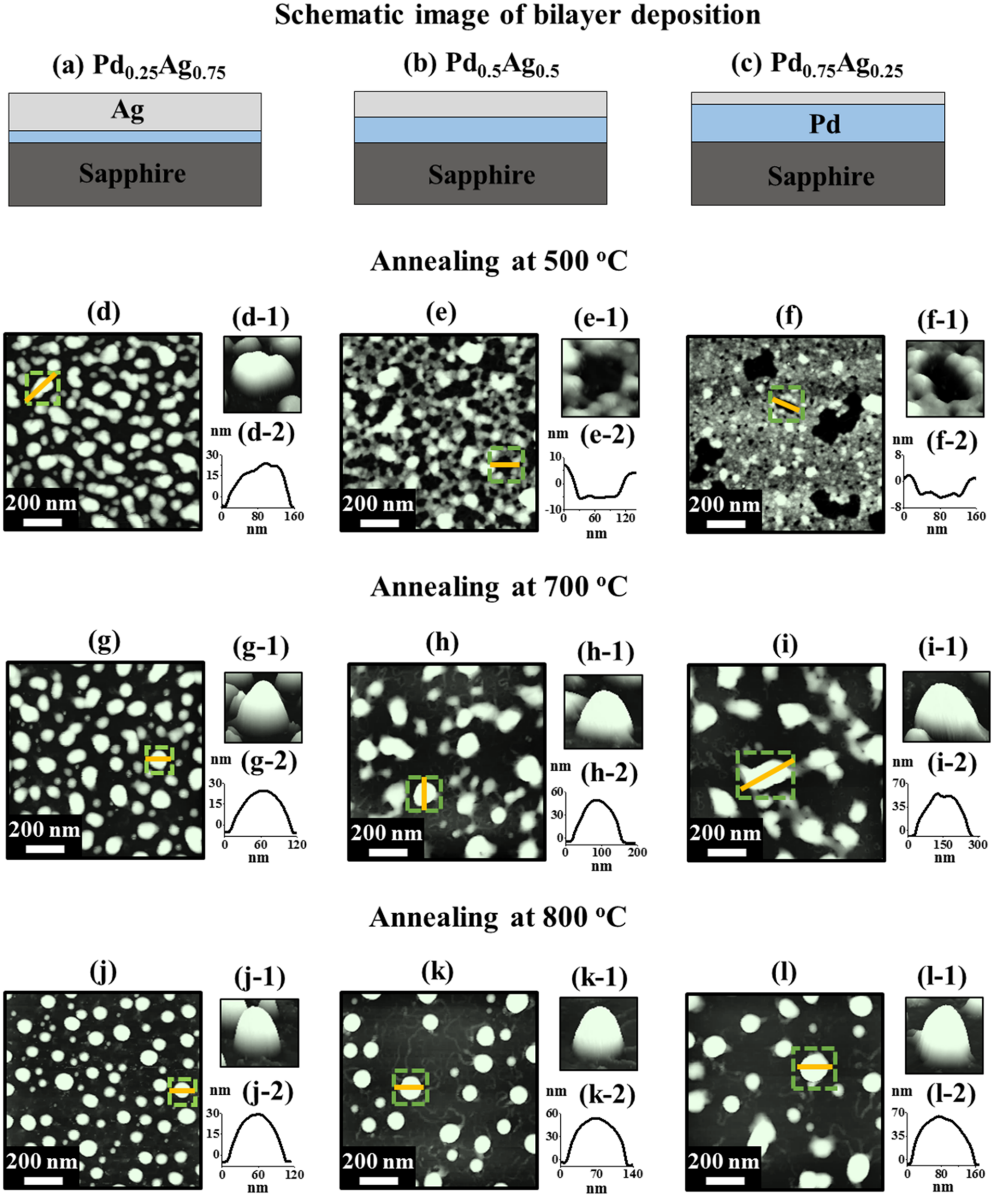
Influence of composition on the evolution of bimetallic Pd-Ag nanostructures at various annealing temperature with a fixed total thickness 6 nm. (a)–(c) Schematics of three different composition of Pd-Ag bilayers, i.e. Pd_0.25_Ag_0.75_, Pd_0.5_Ag_0.5_, Pd_0.75_Ag_0.25_. (d)–(f) From irregular nanoclusters to voids at 500°C. (g)–(i) From alloy NPs to irregular nanoclusters at 700°C. (j)–(l) From small densely packed round dome NPs to large less dense NPs. (d)–(l) AFM top-views of 1 × 1 μm^2^. (d-1)–(l-1) and (d-2)–(l-2) AFM side-views and cross-sectional line-profiles.

Fig 1 shows the evolution of bimetallic Pd-Ag alloy NPs on sapphire (0001) by the control of annealing temperature with a fixed thickness 6 nm and composition Pd_0.5_Ag_0.5_. Corresponding detailed AFM side-views are presented in S2 Fig In general, the formation of voids at low temperature, irregular nanoclusters at intermediate temperature and round dome alloy NPs at high temperature were demonstrated via the solid state dewetting as depicted by the schematic illustrations in Fig 1(A) and AFM images in Fig 1(B)–1(G). A key influential factor in the solid state dewetting of bilayer films can be the surface and inter-diffusion of metallic atoms, which can be initiated at lower energy sites of bilayer films, i.e. steps, grain boundaries, atomic vacancies and defects. At the same time, the substrate surface topography, roughness and the atomic interaction of deposited metal film with substrate can influence the dewetting process of the deposited metallic films [23–28]. The surface roughness of substrate can act as a surface diffusion barrier for metallic atoms, which can hinder the dewetting of metallic film. Since, the sapphire (0001) substrate has a relatively low surface roughness (Ra: ~ 0.101 nm), it can be considered as a good condition for the surface diffusion of metallic atoms. Furthermore, based on the atomic interaction of sapphire with metal films, the surface diffusion of Ag atoms on sapphire is higher as compared to the Pd atoms because of the stronger binding force of Ag-sapphire than the Pd-sapphire [29, 30]. Through the annealing, the surface and inter-diffusion of metallic atoms can be follow the Arrhenius equation, i.e. $D=D_{0}\;exp(-\frac{E_{a}}{kT})$, where D_0_ is the pre-exponential diffusivity, E_a_ is the activation energy of inter-diffusion, T is the temperature, and K is Boltzmann constant [31]. As seen, the diffusivity of atoms is directly determined by the annealing temperature. Along with the diffusion of atoms, the nucleation of tiny pinholes and voids can be initiated in the bilayer films through the coalescence of atomic vacancies as shown in Fig 1(a-ii). Then, the voids can grow by merging the nearby smaller voids along with increased temperature in order to minimize the surface and interface energy and at elevated temperatures, the void coalescence can take place leading to the formation of nanoclusters as shown in Fig 1(a-iii). The growth of voids on Pd/Ag bilayer films can be affected by the activation energy of films [32]. Generally, the activation energies (E_a_) for Pd and Ag are 203 and 200 kJ/mol respectively [33], which indicates a higher surface diffusion of Ag adatoms as E_a_ is in negative exponential. Furthermore, the activation energy of Pd-Ag can vary at various annealing temperature along with the growth of voids. Since, the Pd and Ag have same crystallographic structure, similar lattice parameter and bear no miscibility gap, they are completely miscible at all compositions [22]. Thus, the inter-diffusion of atoms (Pd, Ag) can be significantly enhanced at high temperature and as a result, the alloy NPs can be formed. More specifically, the effect of annealing temperature on the gradual evolution of Pd-Ag alloy NPs is systematically studied by the AFM images, line-profiles, Rq and SAR in Fig 1(B)–1(H). Initially, the mixture of small alloy NPs and voids was resulted by the annealing of Pd_0.5_Ag_0.5_ bilayer (6 nm total thickness) at 400°C for 120 s as shown in Fig 1(B) and 1(b-1). The height of typical alloy NPs was ~ 9 nm and the depth of typical voids was ~ 7 nm as clearly depicted by the cross-sectional line-profile in Fig 1(b-2). Based on the previous studies, well-developed monometallic Ag NPs were formed at 400°C whereas the monometallic Pd films were not dewetted even at 500°C, which can be related to the diffusivity of each material [34, 35]. The formation of alloy NPs on bilayer can be due to the agglomeration of intermixed atoms which diffuse through the pinholes towards the top layer. Furthermore, the void can grow larger with the increased annealing temperature due to the enhanced surface and inter-diffusion of atoms. As a result, the network-like Pd-Ag bimetallic structures were formed at 500°C,, in which the size of void was significantly expanded as shown in Fig 1(C) and 1(c-1). The typical depth of void was increased up to ~ 14 nm as shown in Fig 1(c-2). Eventually at 600 and 700°C, the isolation of elongated bimetallic Pd-Ag nanocluster was demonstrated based on the Rayleigh instability [36] as shown in Fig 1(D) and 1(E). At this temperature, the inter-mixing of Pd and Ag atoms can be significantly enhanced, which can result the formation of well mixed Pd-Ag alloy NPs. At the same time, as clearly seen in the Ag and Pd EDS count plot in Fig 1(I), a slight desorption of Ag atoms was observed due to the sublimation. The Ag count showed a gradual decrease above 500°C and a sharp decrease above 700°C while the Pd count was similar throughout the temperature range. The rate of sublimation of Ag atoms can be related to the following relation [37], R_s_ = (3.513 × 10^22^)(TM_Ag_)^-1/2^P_eq_, where the R_s_, T, M_Ag_ and P_eq_ are the rate of sublimation, annealing temperature, molecular weight of Ag and equilibrium vapor pressure respectively. As the P_eq_ exponentially increases with the T, the rate of sublimation can also increase exponentially. A drastic change in the NP configuration was observed when the annealing temperature was increased to 800°C, which can be due to the extensive sublimation as well as enhanced diffusion. In specific, the round dome shape alloy NPs were formed at 800 and 900°C as shown in Fig 1(F)– 1(f-2). The height of typical NPs was further increased to ~ 56 nm at 800°C and slightly reduce to ~ 40 nm at 900°C. The driving force for the NPs shape transition from the elongated to the round can be due to the minimization of total surface energy in order to attain the most stable configuration [38, 39]. The gradual surface morphology evolution of bimetallic Pd-Ag NPs can also be explained by the Rq and SAR as shown in Fig 1(H) and summarized in S1 Table. At 400°C, the Rq and SAR were 2.3 nm and 1.59% respectively and when the temperature was increased to 800°C, both the Rq and SAR were increased to 11.16 nm and 10.35% respectively. However, they were slightly reduced at 900°C to 10.78 nm and 10.18% due to the NPs size reduction by sublimation as discussed.

**Fig 3:**
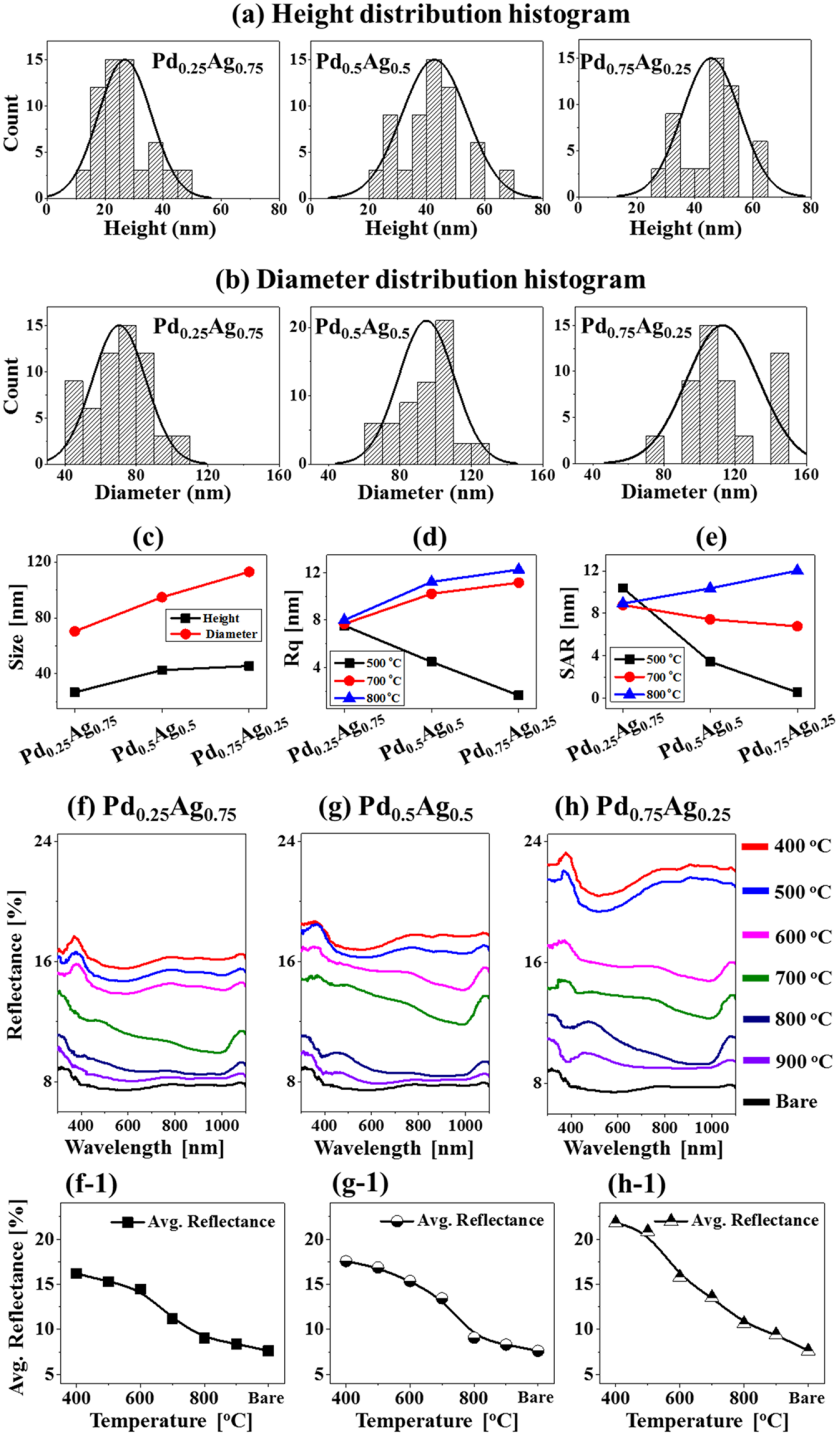
Histograms, morphology parameters and reflectance spectra of annealing temperature sets. (a) Height distribution histogram of Pd-Ag alloy NPs with three different compositions Pd_0.25_Ag_0.75_, Pd_0.5_Ag_0.5_, Pd_0.75_Ag_0.25_ annealed at 800°C for 120 s. (b) Corresponding diameter distribution histogram. (c) Plot of average height and diameter along with the Pd-Ag composition. (d)–(e) Plots of Rq and SAR of various samples annealed at 500, 700 and 800°C. (f)–(h) Reflectance spectra of various Pd-Ag nanostructures for Pd_0.25_Ag_0.75_, Pd_0.5_Ag_0.5_ and Pd_0.75_Ag_0.25_ respectively. (f-1)–(h-1) Plots of corresponding average reflectance versus annealing temperature.

Fig 2 shows the influence of composition on the evolution of various configuration, size, and density of bimetallic Pd-Ag nanostructures. The corresponding surface morphology analysis in terms of size distribution histogram, average height, average diameter, Rq and SAR are presented in Fig 3. The schematics of three compositions of Pd-Ag bilayers (total thickness 6 nm), i.e. Pd_0.25_Ag_0.75_, Pd_0.5_Ag_0.5_, Pd_0.75_Ag_0.25_, are shown in Fig 2(A) and 2(C). In general, during the annealing of various Pd-Ag bilayers, the growth behavior of bimetallic Pd-Ag nanostructures was drastically varied depending on the compositional variation such that the dewetting process of Pd-Ag bilayer films was significantly influenced by the variation of individual thickness of Ag or Pd layers. Since the diffusivity of Pd is lower than Ag, the higher Pd component can suppress the overall dewetting whereas the increased Ag composition can improve the dewetting of Pd-Ag films [40, 41]. For instance, at 500°C with the high Ag content of Pd_0.25_Ag_0.75_, the elongated bimetallic Pd-Ag nanostructures were formed as shown by the AFM top-view in Fig 2(D), magnified side-view in Fig 2(d-1) and line-profile in Fig 2(d-2). With the increased Pd or decreased Ag composition, i.e. for the Pd_0.5_Ag_0.5_ bilayer, the network-like Pd-Ag nanostructures were formed due to the suppression of bilayer film dewetting by the low diffusivity Pd as shown in Fig 2(E) and 2(e-1). When the Pd composition was increased further (Pd_0.75_Ag_0.25_), only few large voids were observed with small vertical size as shown in Fig 2(F)–2(f-2). This indicates a clear suppression of dewetting process by the added Pd component in the bilayer. Similarly, at 700°C, the isolated bimetallic Pd-Ag nanostructures were formed for all three compositions, but the morphology and dimensions were significantly varied as shown in Fig 2(G)–2(I). For instance, co-existence of elongated and round bimetallic NPs for the Pd_0.25_Ag_0.75_, elongated nanostructures for the Pd_0.5_Ag_0.5_ bilayer and connected and elongated nanoclusters for the Pd_0.75_Ag_0.25_ were observed in Fig 2(G)–2(I). At high annealing temperature, i.e. 800°C, the shape of bimetallic NPs was transformed to the round and the size was increased whereas the density was decreased for all compositions as clearly shown in Fig 2(J)–2(L). The curves of height and diameter distribution histograms in Fig 3(A)–3(C) demonstrated a gradually right-shift, indicating an increase in average height and diameter of NPs as discussed. In addition, the changes in the morphology of Pd-Ag nanostructures with different compositions and annealing temperatures can be further explain in terms of the Rq and SAR as shown in Fig 3(D)–3(E) and summarized in S1 Table. At 500°C, the Rq and SAR were gradually decreased in the sequence of Pd_0.25_Ag_0.75_, Pd_0.5_Ag_0.5_ and Pd_0.75_Ag_0.25_ due to the gradual formation of elongated nanostructures, network-like nanoclusters and voids. But at 700°C, the Rq was consistently increased as the vertical size of bimetallic Pd-Ag nanostructures was gradually increased. Meanwhile, the SAR was slightly decreased due to the reduction in density. Likewise, at 800°C, the Rq and SAR of corresponding samples were increased along with the increased size of alloy NPs. Corresponding morphology and EDS analysis of specific bilayer composition with the variation of annealing temperature are presented in S3–S7 Figs. Fig 3(F)–3(H) show the reflectance spectra of corresponding bimetallic Pd-Ag nanostructures with the bilayer thickness 6 nm for the specific bilayer compositions (a) Pd_0.25_Ag_0.75_, (b) Pd_0.5_Ag_0.5_, (c) Pd_0.75_Ag_0.25_. In general, the variant reflectance spectra and average reflectance were observed based on the surface morphology of Pd-Ag nanostructure along with the various composition and annealing temperature. Initially, the reflectance spectrum of bare sapphire exhibited a ~ 8% average reflectance over a range of 300 to 1100 nm, and no obvious peak was observed [39]. By the fabrication of bimetallic Pd-Ag nanostructures, considerable changes in spectral shape and average reflectance was observed. For instance, with the Pd_0.25_Ag_0.75_ annealed between 400 and 600°C, a small peak was observed near ~ 380 nm as shown in Fig 3(F), which can be attributed to the quadrupolar resonance of Pd-Ag nanostructures [42]. At the same time, a wide absorption dip was also observed in VIS region. When the samples were annealed between 700 and 900°C, the quadrupolar resonance peak was vanished and the strong absorption was observed in NIR region along with the formation of isolated NPs. In the case of Pd_0.5_Ag_0.5_ bilayer, between 400 and 500°C, similar reflectance spectra were observed but the intensity was slightly increased because of the higher surface coverage as compared with the Pd_0.25_Ag_0.75_ as shown in Fig 3(G). Between 600 and 900°C, small reflectance peaks were observed in VIS region (~ 475 nm) along with the strong absorption dips in NIR region due to the formation of larger NPs. For the Pd_0.75_Ag_0.25_ set, the dipolar resonance peak was most intensive, and the absorption dip was enhanced and thus an additional reflectance peak in NIR region was observed between 400 and 500°C due to the comparatively wide surface coverage as shown in Fig 3(H). Likewise, the most intense reflectance peak in VIS region was observed for 800 and 900°C along with the formation of larger NPs. For all three compositions, the average reflectance was gradually decreased between 400 and 900°C as shown in Fig 3(f-1)–3(h-1) due to the gradual reduction of surface coverage and sublimation of Ag. Corresponding average reflectance is summarized in S2 Table. (Corresponding Raman spectra are presented in S8 Fig.)

**Fig 4:**
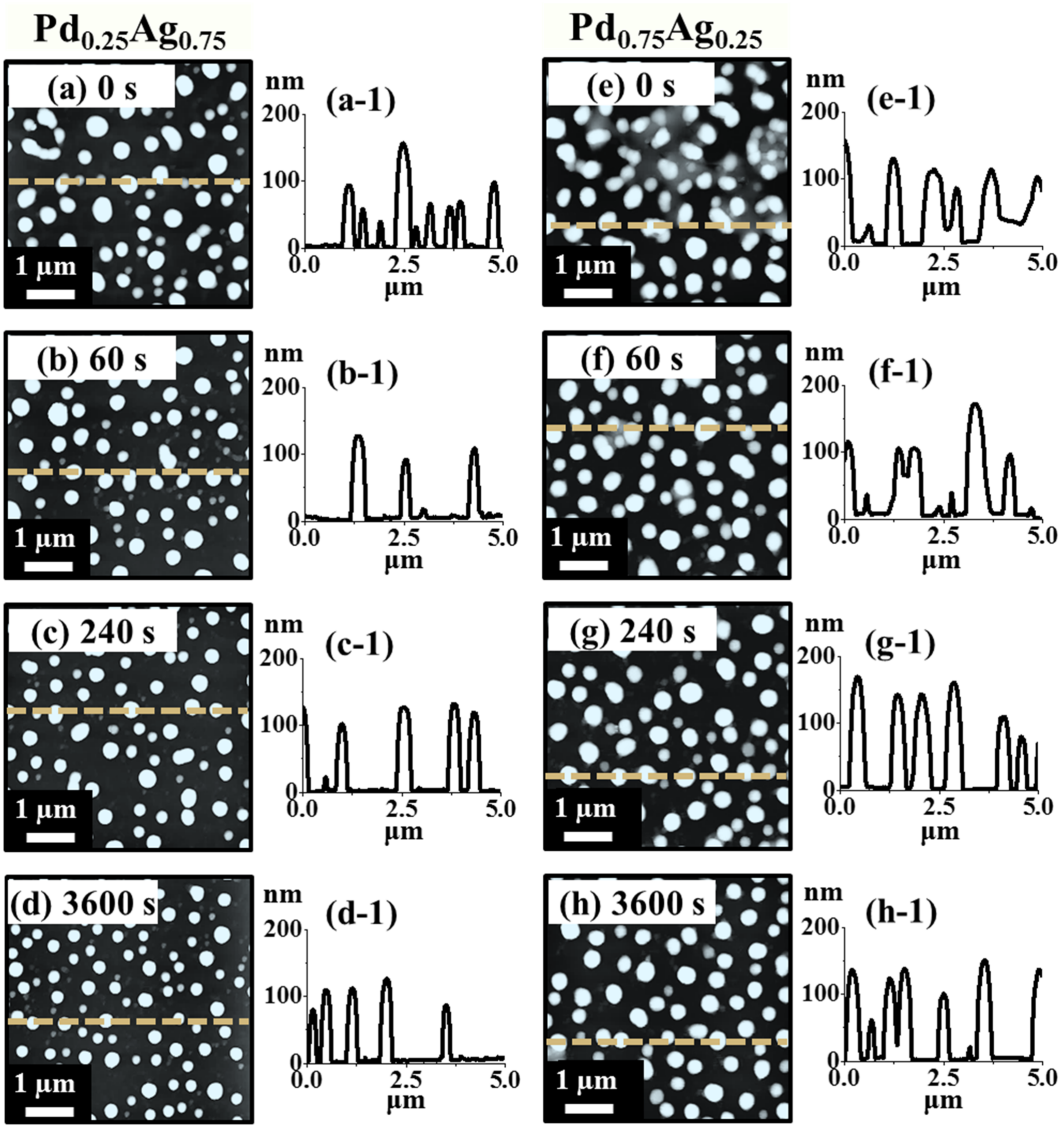
Effect of annealing time on the configuration and size of Pd-Ag alloy NPs with total thickness 20 nm (Pd_0.25_Ag_0.75_ and Pd_0.75_Ag_0.25_) followed by the annealing at 850°C. (a)–(h) AFM top-views (5 × 5 μm^2^) of alloy NPs. (a-1)–(h-1) Corresponding cross-sectional line-profiles.

**Fig 5:**
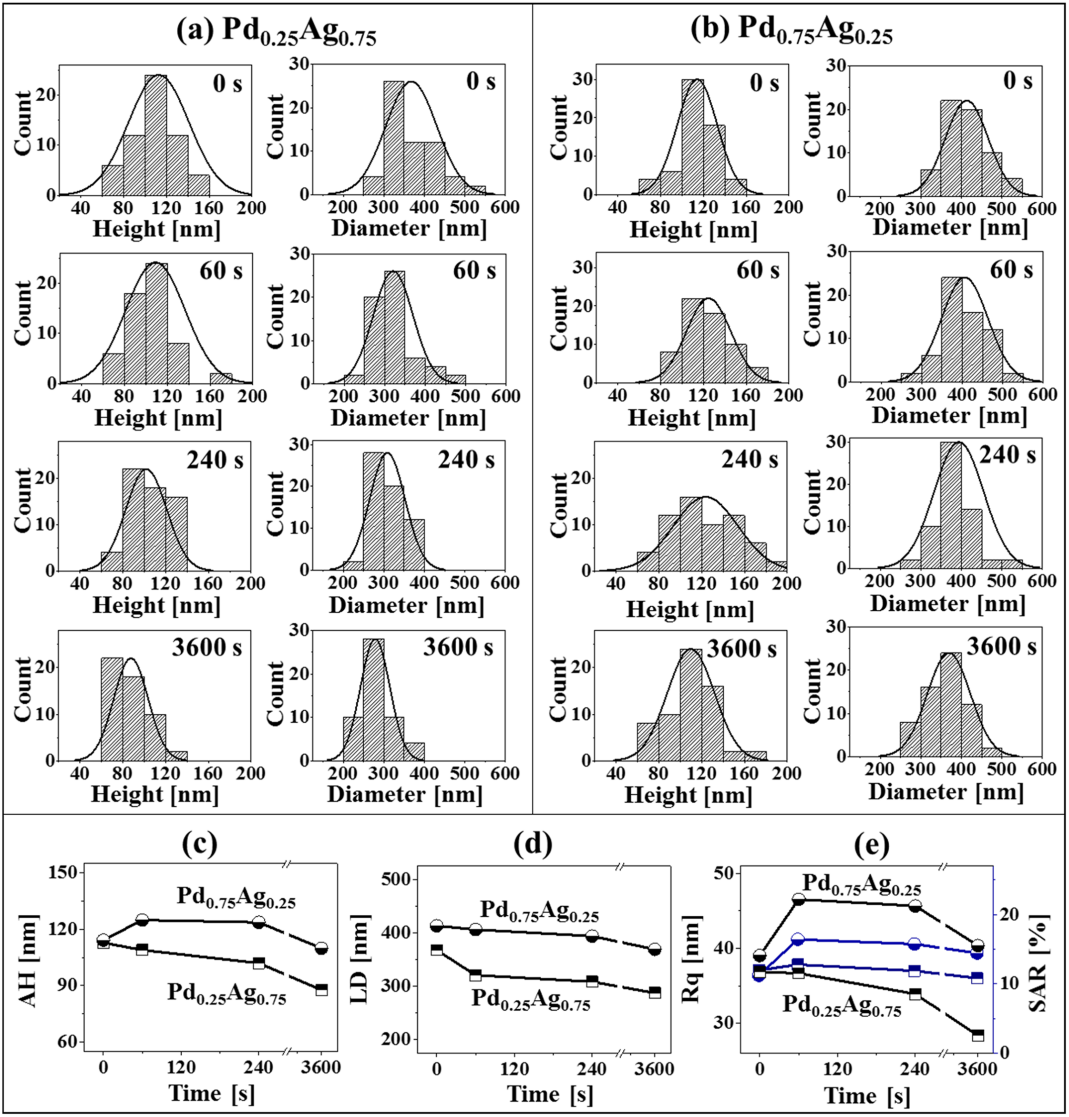
Histograms and morphology parameters for the annealing time sets. (a)–(b) Height and diameter distribution histograms of corresponding Pd-Ag alloy NPs fabricated at various annealing time between 0 and 3600 s with total thickness 20 nm (Pd_0.25_Ag_0.75_ and Pd_0.75_Ag_0.25_). (c)–(d) Plots of average height (AH) and average lateral diameter (LD) of alloy NPs. (e) Plots of Rq and SAR.

Figs 4 and 5 show the formation of various morphologies of Pd-Ag alloy NPs by the control of annealing time at 850°C with the Pd_0.25_Ag_0.75_ and Pd_0.75_Ag_0.25_ compositions (total thickness 20 nm). Corresponding detailed AFM side-views are presented in S9 and S10 Figs. As discussed above, high annealing temperature can enhance the surface and inter-diffusion of Pd and Ag atoms and with the excessive thermal energy, the increase in film thickness can lead to the well-separated larger NPs [43]. With the added thickness of bilayers films at sufficient annealing temperature, the overall self-assembly process can be much enhanced due to the large number of diffusing adatoms. In general, a sharp distinction on the morphology of Pd-Ag alloy NPs was observed between the bilayer compositions within identical annealing time variation between 0 and 3600 s. Both enhanced diffusion and sublimation can occur simultaneously at this temperature of 850°C, which can directly affect the dimension and shape transformation of NPs. For instance, with the Pd_0.25_Ag_0.75_ set, slightly elongated NPs were gradually transformed to the round shape and the size of NPs was steadily decreased with the increased annealing time as shown in Fig 4(A)–4(D). For instance, at 0 s of annealing, elongated and round shaped NPs with wide range of dimension were formed. From the histogram plots, the NPs height and diameter ranged within 60–160 nm and 250–550 nm respectively as shown in Fig 5(A). Along with the increased time, the diffusion and Ag sublimation can be significantly enhanced and therefore, the size of NPs was gradually reduced, and shape became more regular. As shown in the AFM images, line-profiles, histograms and plots in Figs 4(A)–4(D), 5(A) and 5(C)–5(D), the uniformity of NPs was gradually improved, and the average height of NPs was steadily decreased from 112.82 to 87.65 nm whereas the average diameter was rapidly decreased from 367.71 to 287.65 nm between 0 and 3600 s. Whereas with the Pd_0.75_Ag_0.25_, comparatively larger NPs were formed at particular annealing time due to the increased Pd content as compared to the Pd_0.25_Ag_0.75_ set. In specific, the irregular NPs were formed at 0 s due to the higher amount of low diffusivity Pd as shown in Fig 4(E). The surface coverage by the Pd-Ag NPs was higher as compared to the Pd_0.25_Ag_0.75_ composition. Along with the extended annealing time to 60 and 240 s, larger isolated round NPs were observed as clearly seen in Fig 4(F)–4(G). Extension of annealing time to 3600 s led to the formation of well-separated round NPs as evidenced in Fig 4(H). In addition, the histograms of average height and diameter and corresponding plots depict the gradual reduction between 0 and 3600 s along with the sublimation of Ag as shown in Fig 5(B) and 5(C). Similarly, the average diameter was decreased with the increased annealing temperature as shown in Fig 5(D). Furthermore, the changes in the morphology of alloy NPs can also be evidenced by the Rq and SAR of corresponding samples in Fig 5(E) and summarized in S4 Table. Based on the surface morphology with the Pd_0.25_Ag_0.75_ set, the Rq was gradually decreased from 0 to 3600 s but the SAR initially increased from 0 to 60 s likely due to the formation of dense NPs and after that gradually decreased up to 3600 s with the formation of smaller NPs due to the Ag sublimation. On the other hand, both the Rq and SAR were comparatively higher for Pd_0.75_Ag_0.25_ at specific annealing time due to the formation of larger NPs as discussed. (Corresponding analysis of Pd-Ag alloy NPs formation with Pd_0.5_Ag_0.5_ are presented in S11–S13 Figs).

**Fig 6:**
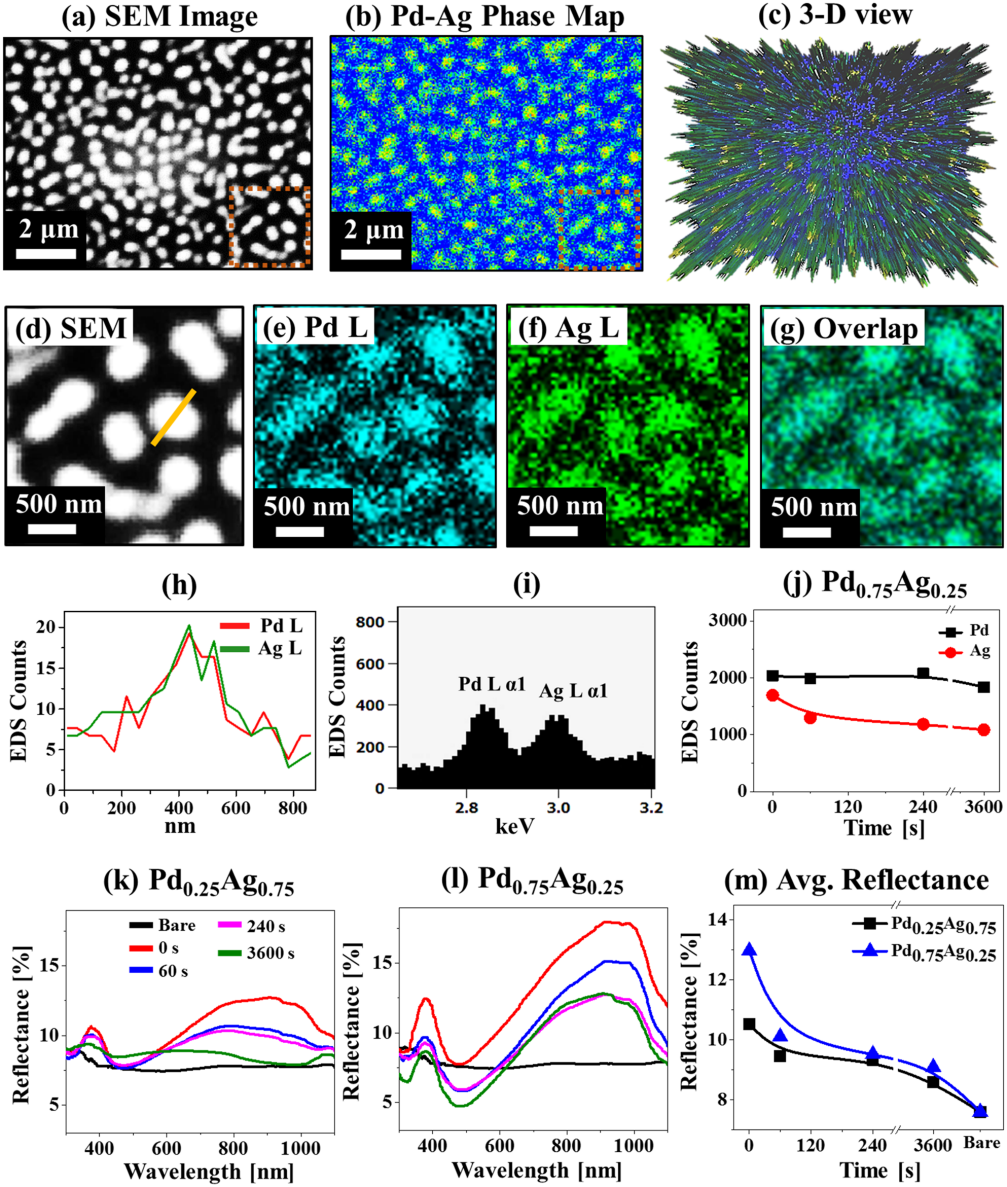
Energy-dispersive x-ray spectroscope (EDS) maps of Pd-Ag alloy NPs fabricated with 20 nm Pd-Ag bilayer thickness with the Pd_0.75_Ag_0.25_ annealed at 850°C for 0 s. (a) SEM image of 10.2 × 7.7 μm^2^. (b) Pd-Ag combined phase map. (c) Corresponding 3-D view. (d) Magnified SEM image (2.5 × 2.5 μm^2^) of particular region marked with the red rectangle in (a). (e)–(g) Corresponding Pd, Ag and overlapped maps. (h) Compositional line-profiles of Pd and Ag. (i) EDS spectrum from the red rectangle in (a). (j) Plot of Pd Lα1 and Ag Lα1 EDS count at various annealing time for the Pd_0.75_Ag_0.25_ set. (k)–(l) Reflectance spectra of Pd-Ag alloy NPs with Pd_0.25_Ag_0.75_ and Pd_0.75_Ag_0.25_ compositions. (m) Plot of average reflectance.

Fig 6(A)–6(J) show the EDS analysis of Pd-Ag alloy NPs with the bilayer thickness of 20 nm (composition Pd_0.75_Ag_0.25_) annealed at 850°C for 0 s. The SEM image is presented in Fig 6(A), Pd-Ag phase map in Fig 6(B) and corresponding 3-D view in Fig 6(C). The SEM image, Pd-Ag phase map and 3-D view were well matched. The typical area elemental analysis of Pd, Ag and overlap maps in Fig 6(D)–6(G) show the formation of homogenous Pd-Ag alloy. In addition, the fabricated NPs shows similar counts of Pd and Ag as supported by the EDS line-profile and EDS spectrum, which can be due to the higher sensitivity of Ag as shown in Fig 6(H)–6(I). Furthermore, the plots of EDS count in Fig 6(J) show the gradual decrement of Ag counts along with the similar Pd counts, indicating a gradual sublimation of Ag atoms as function of time. Fig 6(K)–6(M) show the reflectance spectra of various bimetallic Pd-Ag NPs annealed for different annealing time 0, 60, 240 and 3600 s with the bilayer thickness 20 nm. The reflectance spectra for the Pd_0.25_Ag_0.75_ set is presented in Fig 6(K), which shows the development of quadrupolar resonance peak at ~ 380 nm, absorption dip at ~ 470 nm and strong dipolar resonance peaks at NIR region. The formation of dipolar peaks can be due to the formation of larger NPs as well as plasmonic resonance of Ag because only monometallic Pd NPs did not exhibit this type of spectra [34, 35]. Meanwhile, the magnitude of dipolar peak can be modulated based on the morphology of NPs. For instance, strong reflectance was observed in the spectrum for 0 s with the larger bimetallic NPs and it was gradually reduced with the reduction of NP size for 60 and 240 s, and nearly vanished with the small NPs for 3600 s. In the case of Pd_0.75_Ag_0.25_ set, the reflectance spectra exhibited very strong magnitude of quadrupolar resonance peak, absorption dip and dipolar peak along with the formation of comparatively larger NPs as shown in Fig 6(L). These peaks and dips were equally decreased along with the increased annealing time likely due to the reduction of surface coverage and NPs size. Also, interestingly the absorption dips were lower than the bare sapphire between 60 and 3600 s, which can be due to the enhanced light absorption in the visible region by the larger Pd-Ag alloy NPs. On the other hand, the shift of absorption peak was observed depending upon the NPs size such that it shifted towards longer wavelength based on the increased size and vice-versa [44]. As seen in the reflectance spectra, the 0 s shows absorption dip at ~ 475 nm. With the increased size for 60 s, the absorption dip slightly shifted towards right, i.e. ~ 495 nm and it remains similar for 240 s as the size of NPs was comparable. Then, the size was decreased for 3600 s and thus the absorption dip was shifted towards left i.e. ~ 485 nm. Meanwhile, the dipolar resonance peaks with the Pd_0.75_Ag_0.25_ set was generally right-shifted as compared with the Pd_0.25_Ag_0.75_ c due to the generally larger NP size as discussed. In terms of the average reflectance as shown in Fig 6(M), the average reflectance was gradually decreased for both sets due to the reduction of NP size and surface coverage along with the increased annealing time between 0 and 3600 s. Corresponding average reflectance is summarized in S5 Table. Meanwhile, the Pd_0.75_Ag_0.25_ set showed the slightly enhanced average reflectance at each specific annealing time due to the formation of comparatively larger NPs. (Corresponding Raman spectra are presented in S13 Fig.)

## Conclusion

In conclusion, the investigation on evolution of various morphology of bimetallic Pd-Ag nanostructures was successfully demonstrated on sapphire (0001) via the solid state dewetting of Pd-Ag bilayers. The evolution process of Pd-Ag bimetallic NPs was influenced by the surface and inter-diffusion of constituent atoms, which was modulated by the variation of the annealing temperature, time as well as by the component of Pd and Ag atoms. Due to the gradually enhanced surface and inter-diffusion with the increased annealing temperature, the gradual evolution of voids, porous network, elongated nanoclusters and round alloy NPs were observed. The dewetting process was significantly affected by the composition such that the enhanced overall dewetting was observed for the increased Ag component set and the alloy NPs were formed even at lower annealing temperature. Similarly, the overall dewetting of bilayer was suppressed by the increased Pd content, resulting in the formation of irregular elongated alloy NPs even at high annealing temperature. On the other hand, the control of annealing time between 0 and 3600 s at 850°C resulted in the gradually improved uniformity of alloy NPs with the size reduction owing to the gradual diffusion and desorption of Ag atoms. Furthermore, the reflectance spectra of bimetallic nanostructures demonstrated strong morphology dependent behaviors such that the larger surface coverage exhibited the higher average reflectance and vice-versa. In addition, large NPs with wide-spacing exhibited strong absorption at ~ 470 nm and the absorption dips were red-shifted towards the longer wavelength for the large NPs and vice-versa.

## Supporting information

S1 Fig Surface morphology and optical characteristics of bare sapphire (0001). (a) AFM top-view of bare Sapphire. (b) Corresponding AFM side-view. (c) Cross-sectional line profile. (d) Reflectance spectrum. (e) Raman spectrum.
(DOCX)

S2 Fig AFM side-views (1 × 1 μm^2^) of various Pd-Ag nanostructures: Voids, nanoclusters, NPs by the control annealing temperature between 400 and 900°C for 120 s with composition Pd_0.5_Ag_0.5_ and total thickness 6 nm. (DOCX)

S3 Fig Effect of annealing temperature on the evolution of Pd-Ag alloy NPs on sapphire with 6 nm total thickness (Pd_0.25_Ag_0.75_) and annealing time 120 s. (a)–(f) AFM top-views (1 × 1 μm^2^). (a-1)–(f-1) Cross-sectional line-profiles. (g) Corresponding plot Rq and SAR. (h) Plot of Ag Lα1 and Pd Lα1 EDS count.
(DOCX)

S4 Fig AFM side-views (1 × 1 μm^2^) of various Pd-Ag nanostructures through the systematic variation of annealing temperature with 6 nm total thickness (Pd_0.25_Ag_0.75_) and annealing time 120 s. (DOCX)

S5 Fig Fabrication of various configuration and size of Pd-Ag nanostructures on sapphire by the control of annealing temperature between 400 and 900°C with 6 nm total thickness (Pd_0.75_Ag_0.25_) and annealing time 120 s. (a)–(f) AFM top-views (1 × 1 μm^2^). (a-1)–(f-1) Cross-sectional line-profiles. (g) Plot Rq and SAR. (h) Plot of Ag Lα1 and Pd Lα1 EDS count.
(DOCX)

S6 Fig AFM side-views (1 × 1 μm^2^) of various configuration, size and density of Pd-Ag nanostructures fabricated with 6 nm total thickness (Pd_0.75_Ag_0.25_) and annealing time 120 s. (DOCX)

S7 Fig EDS Spectra of various Pd-Ag nanostructures on sapphire (0001) with fixed thickness 6 nm and different annealing temperature between 400 and 900°C for 120 s. (a) Pd_0.25_Ag_0.75_ (b) Pd_0.5_Ag_0.5_ (c) Pd_0.75_Ag_0.25_.
(DOCX)

S8 Fig (a)–(c) Raman band A_1_g of various Pd-Ag nanostructures on sapphire (0001) with fixed thickness 6 nm and different composition Pd_0.25_Ag_0.75_, Pd_0.5_Ag_0.5_ and Pd_0.75_Ag_0.25_. (d)–(e) Plot of intensity, peak position and FWHM of Raman band A_1_g.
(DOCX)

S9 Fig (a)–(d) AFM side-views (5 × 5 μm^2^) of Pd-Ag nanostructures fabricated with 20 nm bilayer thickness (Pd_0.25_Ag_0.75_) and annealing at 850°C for 0, 60, 240 and 3600 s time. (DOCX)

S10 Fig (a)–(d) AFM side-views (5 × 5 μm^2^) of Pd-Ag nanostructure on sapphire (0001) with various annealing time 0, 60, 240 and 3600 s. The total thickness, composition and annealing temperature were fixed at 20 nm, Pd_0.75_Ag_0.25_ and 850°**C** respectively.
(DOCX)

S11 Fig Various Pd-Ag alloy NPs by the variation of annealing duration with a composition (Pd_0.5_Ag_0.5_) and annealing at 850°C. (a)–(d) AFM top-views of 5 × 5 μm^2^. (a-1)–(d-1) Enlarged AFM side-view (1 × 1 μm^2^) and cross-sectional line-profiles. (e) Plots of Rq and SAR. (f)–(g) Corresponding reflectance spectra and average reflectance.
(DOCX)

S12 Fig (a)–(d) AFM side-views (5 × 5 μm^2^) of various Pd-Ag nanostructures fabricated with 20 nm bilayer thickness (Pd_0.5_Ag_0.5_). The annealing temperature was fixed at 850°C and the time was systematically varied between 0 and 3600 s.
(DOCX)

S13 Fig (a)–(c) Raman spectra of Pd-Ag nanostructures on sapphire (0001) with a fixed thickness 20 nm, annealing temperature 850°C for particular compositions of Pd_0.25_Ag_0.75_, Pd_0.5_Ag_0.5_ and Pd_0.75_Ag_0.25_. (d)–(e) Plot of intensity, peak position and FWHM of Raman band A1g.
(DOCX)

S1 Table Summary of Rq and SAR of various Pd-Ag nanostructures on sapphire (0001) by the control of Pd-Ag compsotion and annealing temperature with total thickness of 6 nm. (DOCX)

S2 Table Summary of average reflectance of Pd-Ag nanostructures with 6 nm total thickness followed by the annealing at various temperature with different Pd-Ag compositions. (DOCX)

S3 Table Summary of Raman intensity (peak counts), peak position and FWHM of Raman band A1g of various Pd-Ag nanostructures by the annealing at various temperature with fixed total thickness 6 nm and different Pd-Ag compositions. (DOCX)

S4 Table Summary of Rq and SAR of various Pd-Ag nanostructures on with various Pd-Ag composition and annealing time at fixed annealing temperature 850°C and total thickness 20 nm. (DOCX)

S5 Table Summary of average reflectance of Pd-Ag nanostructures with 20 nm total thickness with different Pd-Ag compositions and with the variation of annealing time at 850°C. (DOCX)

S6 Table Summary of intensity (peak counts), peak position and FWHM of Raman band A1g of various Pd-Ag nanostructures fabricated with fixed total thickness 20 nm and annealing temperature 850°C. (DOCX)
