# Supplementary material for: Investigation on the morphological and optical evolution of bimetallic Pd-Ag nanoparticles on sapphire (0001) by the systematic control of composition, annealing temperature and time
Source: PLoS One. 2017 Dec 18;12(12):e0189823. doi: 10.1371/journal.pone.0189823 (PMC5734721; doi:10.1371/journal.pone.0189823)
Supplement: S1 Fig — (a) AFM top-view of bare Sapphire. (b) Corresponding AFM side-view. (c) Cross-sectional line profile. (d) Reflectance spectrum. (e) Raman spectrum. (DOCX) [file pone.0189823.s001.docx]

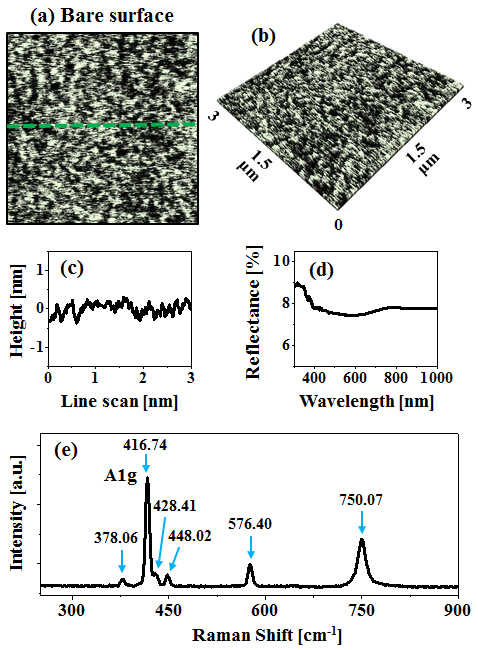


**S1 Fig.** Surface morphology and optical characteristics of bare sapphire (0001). (a) AFM top-view of bare Sapphire. (b) Corresponding AFM side-view. (c) Cross-sectional line profile. (d) Reflectance spectrum. (e) Raman spectrum.
