# Supplementary material for: Investigation on the morphological and optical evolution of bimetallic Pd-Ag nanoparticles on sapphire (0001) by the systematic control of composition, annealing temperature and time
Source: PLoS One. 2017 Dec 18;12(12):e0189823. doi: 10.1371/journal.pone.0189823 (PMC5734721; doi:10.1371/journal.pone.0189823)
Supplement: S2 Fig — (DOCX) [file pone.0189823.s002.docx]

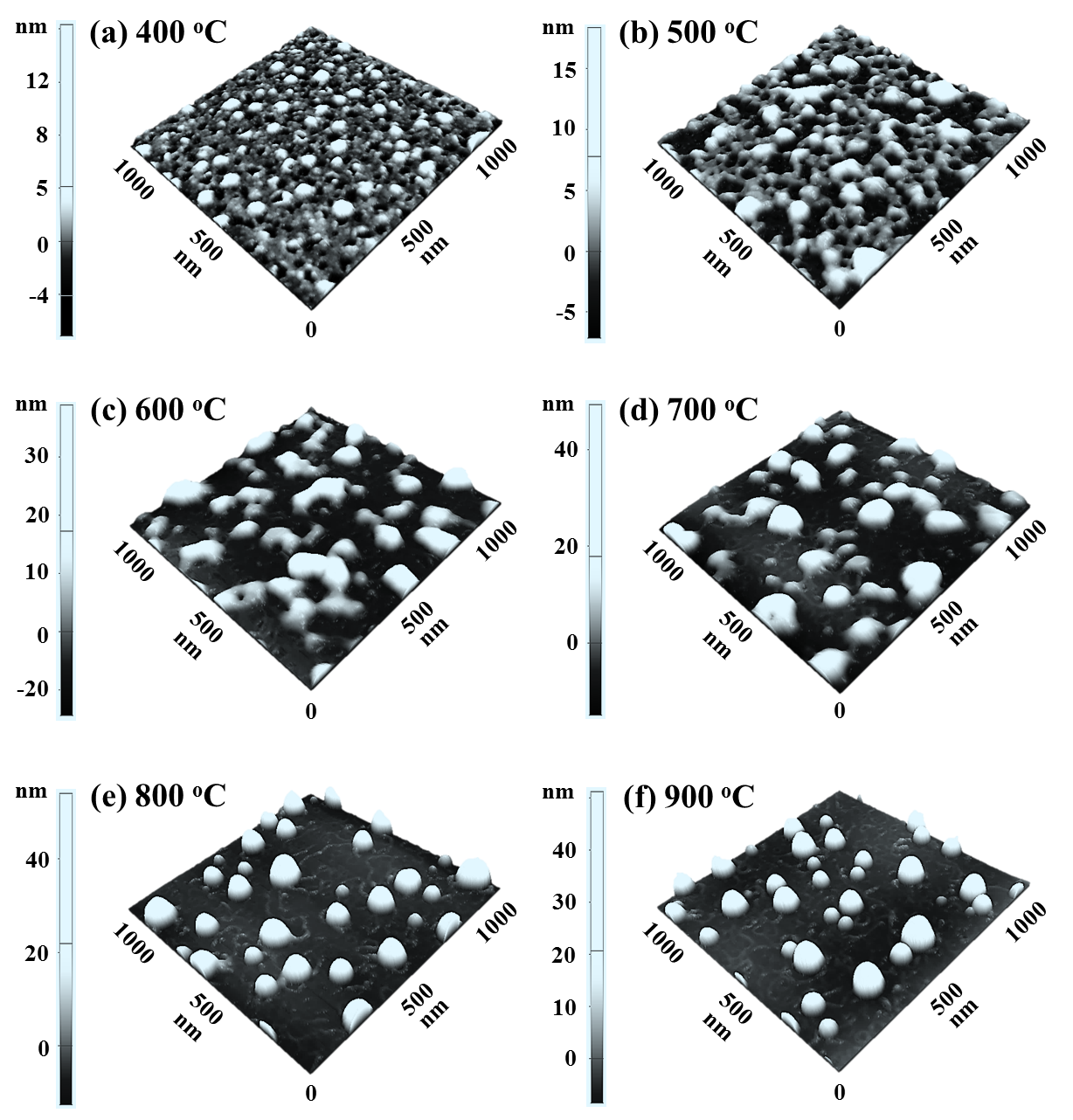


**S2 Fig.** AFM side-views (1 × 1 µm^2^) of various Pd-Ag nanostructures: voids, nanoclusters, NPs by the control annealing temperature between 400 and 900 ^o^C for 120 s with composition Pd_0.5_Ag_0.5_ and total thickness 6 nm.
