# Supplementary material for: Investigation on the morphological and optical evolution of bimetallic Pd-Ag nanoparticles on sapphire (0001) by the systematic control of composition, annealing temperature and time
Source: PLoS One. 2017 Dec 18;12(12):e0189823. doi: 10.1371/journal.pone.0189823 (PMC5734721; doi:10.1371/journal.pone.0189823)
Supplement: S5 Fig — (a)–(f) AFM top-views (1 × 1 μm2). (a-1)–(f-1) Cross-sectional line-profiles. (g) Plot Rq and SAR. (h) Plot of Ag Lα1 and Pd Lα1 EDS count. (DOCX) [file pone.0189823.s005.docx]

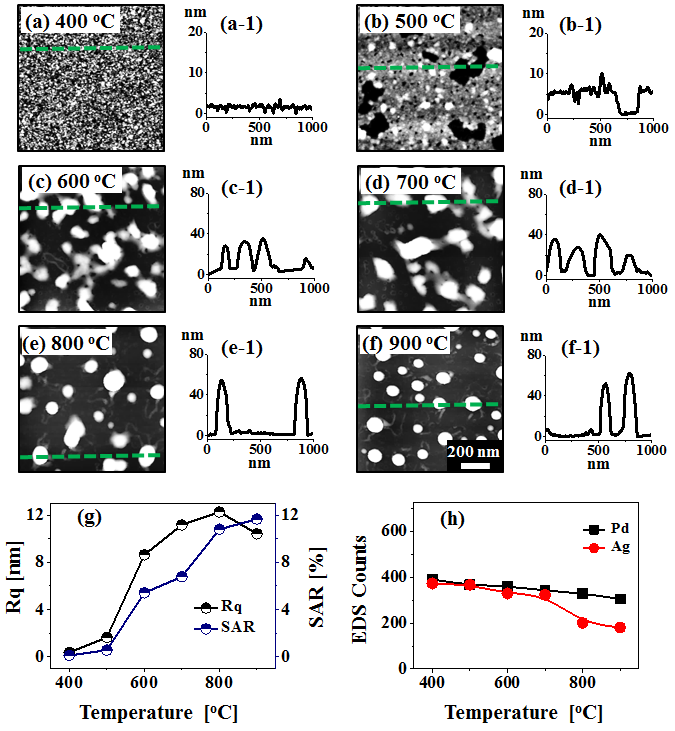


**S5 Fig.** Fabrication of various configuration and size of Pd-Ag nanostructures on sapphire by the control of annealing temperature between 400 and 900 ^o^C with 6 nm total thickness (Pd_0.75_Ag_0.25_) and annealing time 120 s. (a) – (f) AFM top-views (1 × 1 µm^2^). (a-1) – (f-1) Cross-sectional line-profiles. (g) Plot Rq and SAR. (h) Plot of Ag Lα1 and Pd Lα1 EDS count.
