# Supplementary material for: Investigation on the morphological and optical evolution of bimetallic Pd-Ag nanoparticles on sapphire (0001) by the systematic control of composition, annealing temperature and time
Source: PLoS One. 2017 Dec 18;12(12):e0189823. doi: 10.1371/journal.pone.0189823 (PMC5734721; doi:10.1371/journal.pone.0189823)
Supplement: S6 Fig — (DOCX) [file pone.0189823.s006.docx]

**
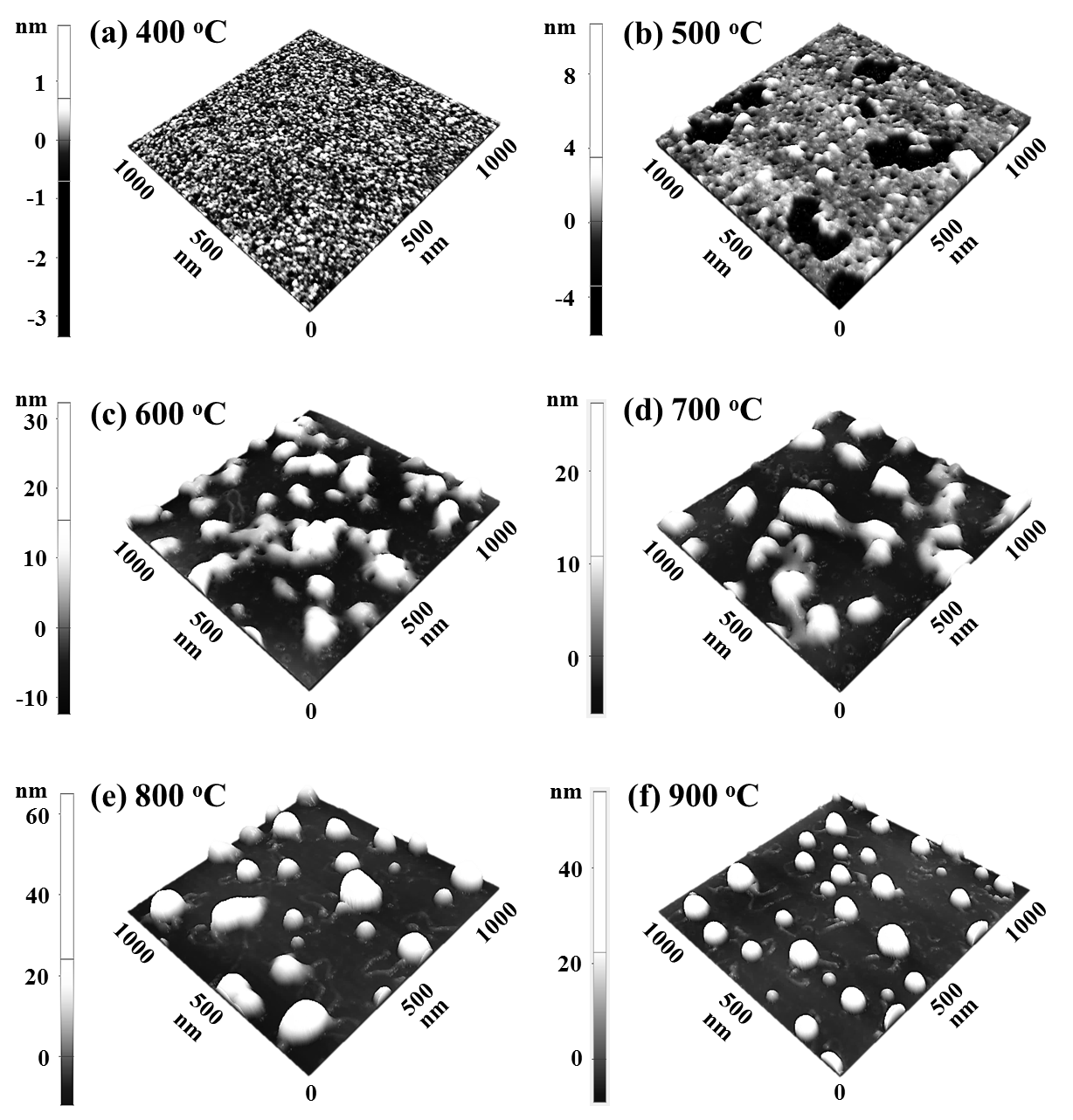
**

**S6 Fig.** AFM side-views (1 × 1 µm^2^) of various configuration, size and density of Pd-Ag nanostructures fabricated with 6 nm total thickness (Pd_0.75_Ag_0.25_) and annealing time 120 s.
