# Supplementary material for: Investigation on the morphological and optical evolution of bimetallic Pd-Ag nanoparticles on sapphire (0001) by the systematic control of composition, annealing temperature and time
Source: PLoS One. 2017 Dec 18;12(12):e0189823. doi: 10.1371/journal.pone.0189823 (PMC5734721; doi:10.1371/journal.pone.0189823)
Supplement: S7 Fig — (a) Pd0.25Ag0.75 (b) Pd0.5Ag0.5 (c) Pd0.75Ag0.25. (DOCX) [file pone.0189823.s007.docx]

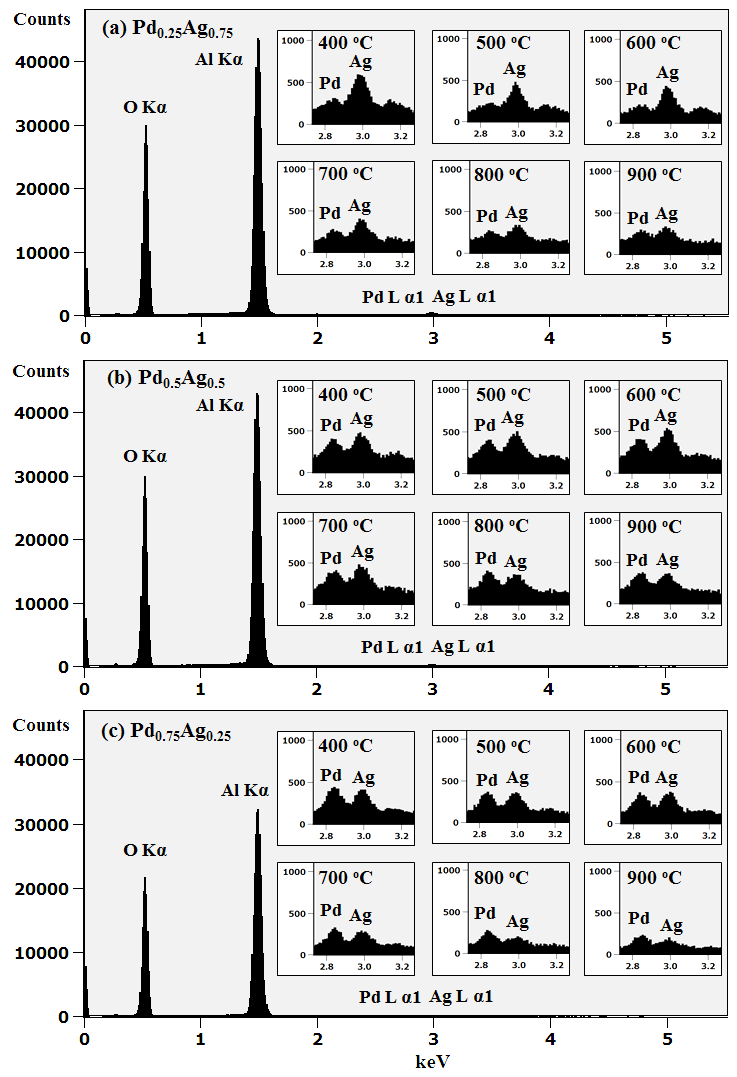


**S7 Fig.** EDS Spectra of various Pd-Ag nanostructures on sapphire (0001) with fixed thickness 6 nm and different annealing temperature between 400 and 900 ^o^C for 120 s. (a) Pd_0.25_Ag_0.75_ (b) Pd_0.5_Ag_0.5_ (c) Pd_0.75_Ag_0.25_.
