# Supplementary material for: Investigation on the morphological and optical evolution of bimetallic Pd-Ag nanoparticles on sapphire (0001) by the systematic control of composition, annealing temperature and time
Source: PLoS One. 2017 Dec 18;12(12):e0189823. doi: 10.1371/journal.pone.0189823 (PMC5734721; doi:10.1371/journal.pone.0189823)
Supplement: S10 Fig — The total thickness, composition and annealing temperature were fixed at 20 nm, Pd0.75Ag0.25 and 850°C respectively. (DOCX) [file pone.0189823.s010.docx]

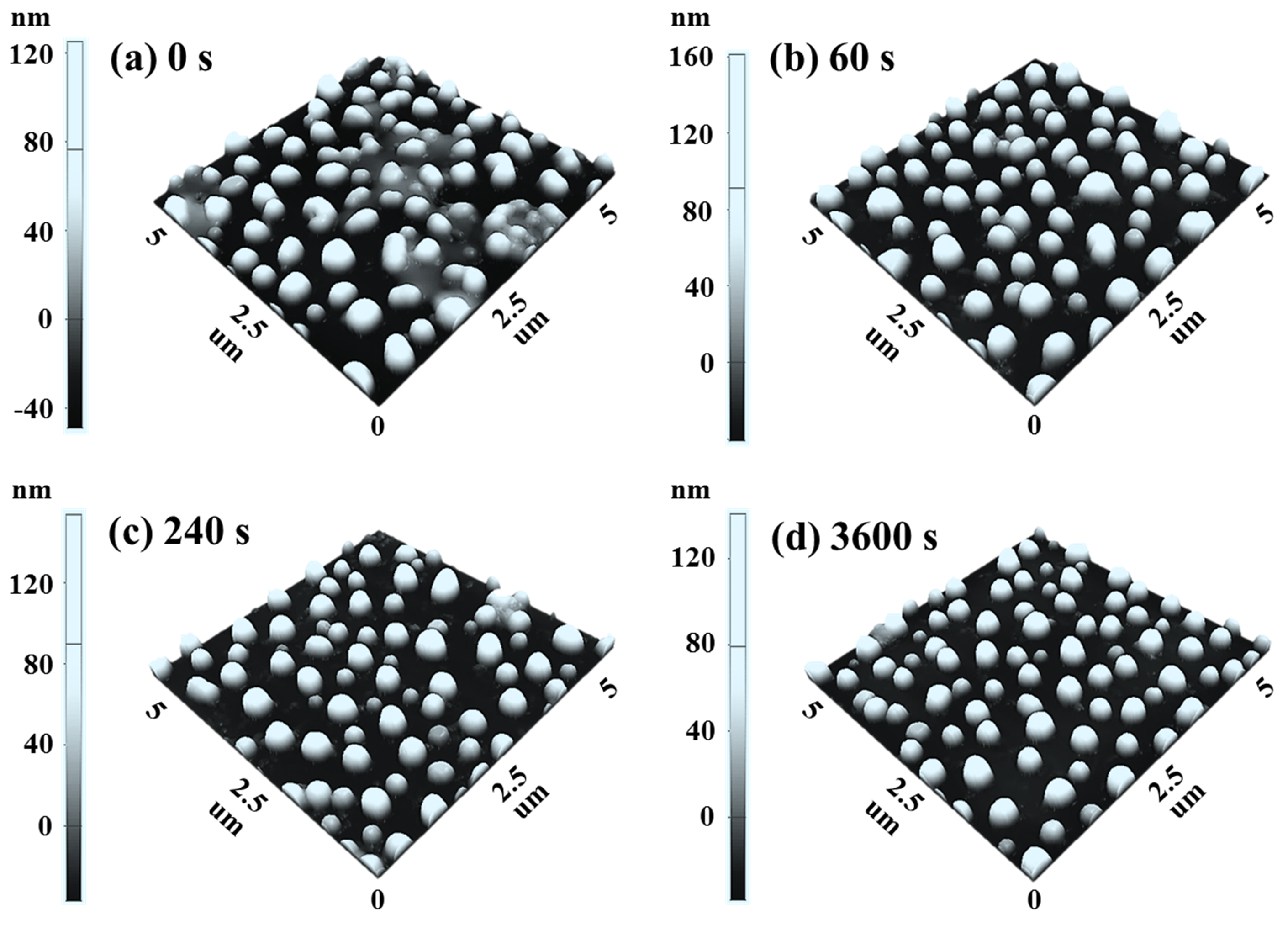


**S10 Fig.** (a) – (d) AFM side-views (5 × 5 µm^2^) of Pd-Ag nanostructure on sapphire (0001) with various annealing time 0, 60, 240 and 3600 s. The total thickness, composition and annealing temperature were fixed at 20 nm, Pd_0.75_Ag_0.25_ and 850 ^o^C respectively.
