# Supplementary material for: Investigation on the morphological and optical evolution of bimetallic Pd-Ag nanoparticles on sapphire (0001) by the systematic control of composition, annealing temperature and time
Source: PLoS One. 2017 Dec 18;12(12):e0189823. doi: 10.1371/journal.pone.0189823 (PMC5734721; doi:10.1371/journal.pone.0189823)
Supplement: S11 Fig — (a)–(d) AFM top-views of 5 × 5 μm2. (a-1)–(d-1) Enlarged AFM side-view (1 × 1 μm2) and cross-sectional line-profiles. (e) Plots of Rq and SAR. (f)–(g) Corresponding reflectance spectra and average reflectance. (DOCX) [file pone.0189823.s011.docx]

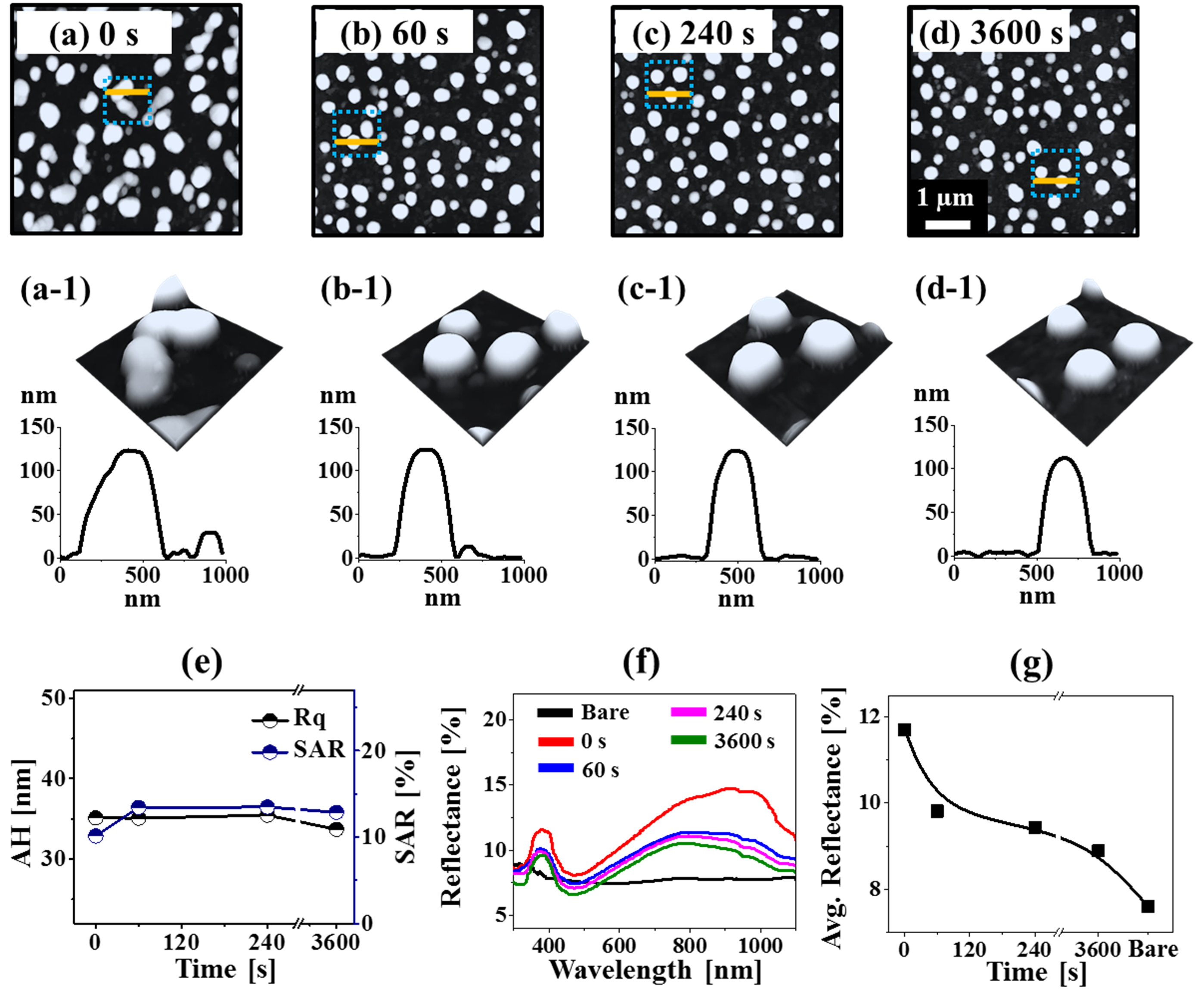


**S11 Fig.** Various Pd-Ag alloy NPs by the variation of annealing duration with a composition (Pd_0.5_Ag_0.5_) and annealing at 850 ^o^C. (a) – (d) AFM top-views of 5 × 5 µm^2^. (a-1) – (d-1) Enlarged AFM side-view (1 × 1 µm^2^) and cross-sectional line-profiles. (e) Plots of Rq and SAR. (f) – (g) Corresponding reflectance spectra and average reflectance.
