# Supplementary material for: Investigation on the morphological and optical evolution of bimetallic Pd-Ag nanoparticles on sapphire (0001) by the systematic control of composition, annealing temperature and time
Source: PLoS One. 2017 Dec 18;12(12):e0189823. doi: 10.1371/journal.pone.0189823 (PMC5734721; doi:10.1371/journal.pone.0189823)
Supplement: S12 Fig — The annealing temperature was fixed at 850°C and the time was systematically varied between 0 and 3600 s. (DOCX) [file pone.0189823.s012.docx]

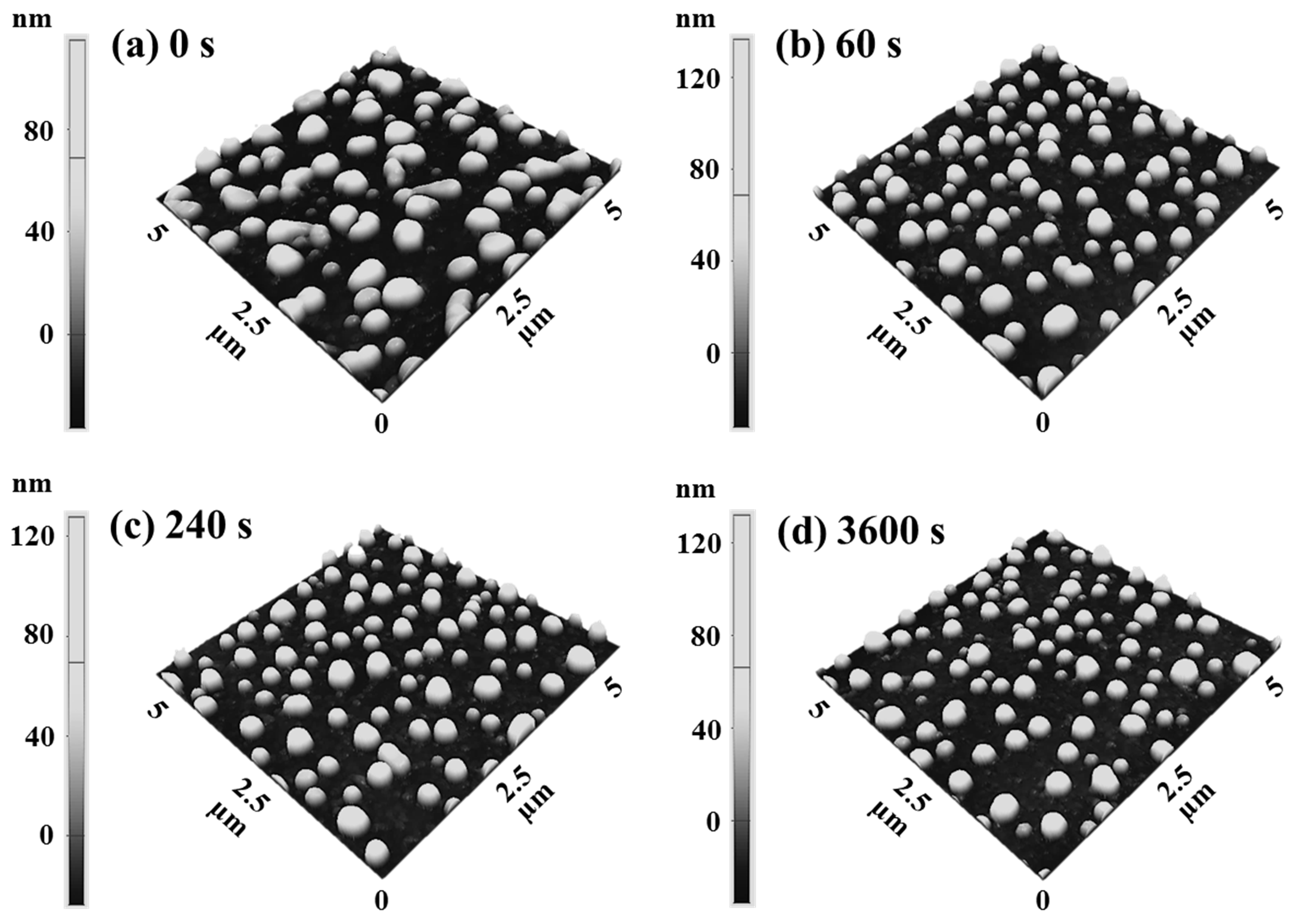


**S12 Fig.** (a) – (d) AFM side-views (5 × 5 µm^2^) of various Pd-Ag nanostructures fabricated with 20 nm bilayer thickness (Pd_0.5_Ag_0.5_). The annealing temperature was fixed at 850 ^o^C and the time was systematically varied between 0 and 3600 s.
