# Supplementary material for: Investigation on the morphological and optical evolution of bimetallic Pd-Ag nanoparticles on sapphire (0001) by the systematic control of composition, annealing temperature and time
Source: PLoS One. 2017 Dec 18;12(12):e0189823. doi: 10.1371/journal.pone.0189823 (PMC5734721; doi:10.1371/journal.pone.0189823)
Supplement: S13 Fig — (a)–(c) Raman spectra of Pd-Ag nanostructures on sapphire (0001) with a fixed thickness 20 nm, annealing temperature 850°C for particular compositions of Pd0.25Ag0.75, Pd0.5Ag0.5 and Pd0.75Ag0.25. (d)–(e) Plot of intensity, peak position and FWHM of Raman band A1g. (DOCX) [file pone.0189823.s013.docx]

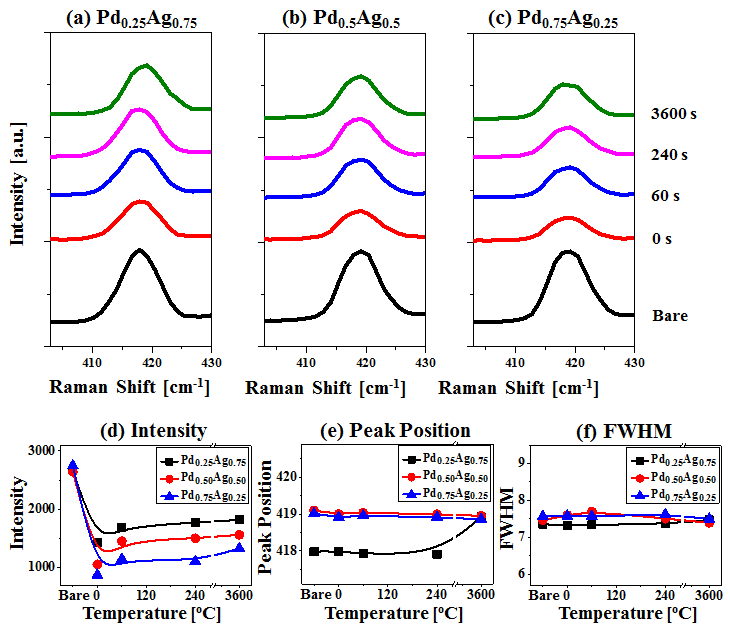


**S13 Fig.** (a) – (c) Raman spectra of Pd-Ag nanostructures on sapphire (0001) with a fixed thickness 20 nm, annealing temperature 850 ^o^C for particular compositions of Pd_0.25_Ag_0.75_, Pd_0.5_Ag_0.5_ and Pd_0.75_Ag_0.25_. (d) – (e) Plot of intensity, peak position and FWHM of Raman band A_1_g.
