# Supplementary material for: Investigation on the morphological and optical evolution of bimetallic Pd-Ag nanoparticles on sapphire (0001) by the systematic control of composition, annealing temperature and time
Source: PLoS One. 2017 Dec 18;12(12):e0189823. doi: 10.1371/journal.pone.0189823 (PMC5734721; doi:10.1371/journal.pone.0189823)
Supplement: S1 Table — (DOCX) [file pone.0189823.s014.docx]

**S1 Table.** Summary of Rq and SAR of various Pd-Ag nanostructures on sapphire (0001) by the control of Pd-Ag compsotion and annealing temperature with total thickness of 6 nm.

|  | **Pd_0.25_Ag_0.75_** | | **Pd_0.5_Ag_0.5_** | | **Pd_0.75_Ag_0.25_** | |
| --- | --- | --- | --- | --- | --- | --- |
| **Temperature [^o^C]** | **Rq [nm]** | **SAR [%]** | **Rq [nm]** | **SAR [%]** | **Rq [nm]** | **SAR [%]** |
| **400** | 4.19 | 2.38 | 2.3 | 1.59 | 0.35 | 0.10 |
| **500** | 7.53 | 10.38 | 4.47 | 3.44 | 1.63 | 0.55 |
| **600** | 9.12 | 12.01 | 9.27 | 7.14 | 8.63 | 5.41 |
| **700** | 7.68 | 8.78 | 10.22 | 7.43 | 11.16 | 6.78 |
| **800** | 7.99 | 9.50 | 11.16 | 10.35 | 12.26 | 12.02 |
| **900** | 7.54 | 8.93 | 10.78 | 10.18 | 12.41 | 11.66 |
