# Supplementary material for: Investigation on the morphological and optical evolution of bimetallic Pd-Ag nanoparticles on sapphire (0001) by the systematic control of composition, annealing temperature and time
Source: PLoS One. 2017 Dec 18;12(12):e0189823. doi: 10.1371/journal.pone.0189823 (PMC5734721; doi:10.1371/journal.pone.0189823)
Supplement: S2 Table — (DOCX) [file pone.0189823.s015.docx]

**S2 Table.** Summary of average reflectance of Pd-Ag nanostructures with 6 nm total thickness followed by the annealing at various temperature with different Pd-Ag compositions.

| **Reflectance Summary [%]** | | | |
| --- | --- | --- | --- |
| **Time [s]** | **Pd_0.25_Ag_0.75_** | **Pd_0.5_Ag_0.5_** | **Pd_0.75_Ag_0.25_** |
| **Bare** | 7.60 | 7.62 | 7.62 |
| **400** | 15.30 | 17.55 | 21.8 |
| **500** | 14.45 | 16.85 | 20.82 |
| **600** | 11.17 | 15.34 | 15.80 |
| **700** | 9.03 | 13.44 | 13.54 |
| **800** | 8.41 | 9.07 | 10.64 |
| **900** | 7.62 | 8.32 | 9.42 |
