# Supplementary material for: Investigation on the morphological and optical evolution of bimetallic Pd-Ag nanoparticles on sapphire (0001) by the systematic control of composition, annealing temperature and time
Source: PLoS One. 2017 Dec 18;12(12):e0189823. doi: 10.1371/journal.pone.0189823 (PMC5734721; doi:10.1371/journal.pone.0189823)
Supplement: S3 Table — (DOCX) [file pone.0189823.s016.docx]

**S3 Table.** Summary of Raman intensity (peak counts), peak position and FWHM of Raman band A1g of various Pd-Ag nanostructures by the annealing at various temperature with fixed total thickness 6 nm and different Pd-Ag compositions.

| **(a)** | **Raman Summary (Pd_0.25_Ag_0.75_)** | | |
| --- | --- | --- | --- |
| **Temperature [^o^C]** | **Peak Counts** | **Peak Position** | **FWHM** |
| **Bare** | 2610.27 | 416.74 | 6.98 |
| **400** | 1278.11 | 416.50 | 7.02 |
| **500** | 1318.13 | 417.18 | 7.30 |
| **600** | 1308.79 | 417.28 | 7.33 |
| **700** | 1675.05 | 417.32 | 7.32 |
| **800** | 2326.33 | 417.23 | 7.08 |
| **900** | 2499.43 | 417.12 | 7.11 |
| **(b)** | **Raman Summary (Pd_0.5_Ag_0.5_)** | | |
| **Temperature [^o^C]** | **Peak Counts** | **Peak Position** | **FWHM** |
| **Bare** | 2610.27 | 416.74 | 6.98 |
| **400** | 1185.89 | 416.06 | 7.11 |
| **500** | 1174.25 | 416.13 | 7.06 |
| **600** | 1199.29 | 416.07 | 7.05 |
| **700** | 1384.29 | 416.35 | 7.57 |
| **800** | 1978.31 | 416.26 | 7.31 |
| **900** | 2164.08 | 416.23 | 7.35 |
| **(c)** | **Raman Summary (Pd_0.75_Ag_0.25_)** | | |
| **Temperature [^o^C]** | **Peak Counts** | **Peak Position** | **FWHM** |
| **Bare** | 2610.27 | 416.74 | 6.98 |
| **400** | 936.28 | 417.20 | 7.39 |
| **500** | 884.62 | 417.24 | 7.38 |
| **600** | 1213.81 | 417.26 | 7.32 |
| **700** | 1174.05 | 417.16 | 7.23 |
| **800** | 1706.91 | 417.19 | 7.24 |
| **900** | 2107.71 | 417.22 | 7.27 |
