# Supplementary material for: Investigation on the morphological and optical evolution of bimetallic Pd-Ag nanoparticles on sapphire (0001) by the systematic control of composition, annealing temperature and time
Source: PLoS One. 2017 Dec 18;12(12):e0189823. doi: 10.1371/journal.pone.0189823 (PMC5734721; doi:10.1371/journal.pone.0189823)
Supplement: S4 Table — (DOCX) [file pone.0189823.s017.docx]

**S4 Table.** Summary of Rq and SAR of various Pd-Ag nanostructures on with various Pd-Ag composition and annealing time at fixed annealing temperature 850 ^o^C and total thickness 20 nm.

|  | **Pd_0.25_Ag_0.75_** | | **Pd_0.5_Ag_0.5_** | | **Pd_0.75_Ag_0.25_** | |
| --- | --- | --- | --- | --- | --- | --- |
| **Time [s]** | **Rq [nm]** | **SAR [%]** | **Rq [nm]** | **SAR [%]** | **Rq [nm]** | **SAR [%]** |
| **0** | 36.86 | 11.95 | 35.13 | 10.13 | 39.03 | 11.22 |
| **60** | 36.67 | 12.78 | 35.09 | 13.44 | 46.54 | 16.41 |
| **240** | 33.91 | 11.87 | 35.47 | 13.47 | 45.66 | 15.75 |
| **3600** | 28.36 | 10.83 | 33.71 | 12.87 | 40.38 | 14.44 |
