# Supplementary material for: Investigation on the morphological and optical evolution of bimetallic Pd-Ag nanoparticles on sapphire (0001) by the systematic control of composition, annealing temperature and time
Source: PLoS One. 2017 Dec 18;12(12):e0189823. doi: 10.1371/journal.pone.0189823 (PMC5734721; doi:10.1371/journal.pone.0189823)
Supplement: S5 Table — (DOCX) [file pone.0189823.s018.docx]

**S5 Table.** Summary of average reflectance of Pd-Ag nanostructures with 20 nm total thickness with different Pd-Ag compositions and with the variation of annealing time at 850 ^o^C.

| **Reflectance Summary [%]** | | | | |
| --- | --- | --- | --- | --- |
| **Time [s]** | **Pd_0.25_Ag_0.75_** | **Pd_0.5_Ag_0.5_** | **Pd_0.75_Ag_0.25_** |  |
| **Bare** | 7.60 | 7.60 | 7.60 |  |
| **0** | 10.53 | 11.70 | 12.97 |  |
| **60** | 9.46 | 9.81 | 10.10 |  |
| **240** | 9.33 | 9.43 | 9.52 |  |
| **3600** | 8.59 | 8.89 | 9.08 |  |
