# Supplementary material for: Investigation on the morphological and optical evolution of bimetallic Pd-Ag nanoparticles on sapphire (0001) by the systematic control of composition, annealing temperature and time
Source: PLoS One. 2017 Dec 18;12(12):e0189823. doi: 10.1371/journal.pone.0189823 (PMC5734721; doi:10.1371/journal.pone.0189823)
Supplement: S6 Table — (DOCX) [file pone.0189823.s019.docx]

**S6 Table.** Summary of intensity (peak counts), peak position and FWHM of Raman band A1g of various Pd-Ag nanostructures fabricated with fixed total thickness 20 nm and annealing temperature 850 ^o^C.

| **(a)** | **Raman Summary (Pd_0.25_Ag_0.75_)** | | | |
| --- | --- | --- | --- | --- |
| **Time [s]** | | **Peak Counts** | **Peak Position** | **FWHM** |
| **Bare** | | 2674.31 | 417.98 | 7.35 |
| **0** | | 1433.95 | 417.99 | 7.32 |
| **60** | | 1690.45 | 417.92 | 7.33 |
| **240** | | 1773.25 | 417.90 | 7.36 |
| **3600** | | 18.17.66 | 418.92 | 7.46 |
| **(b)** | **Raman Summary (Pd_0.5_Ag_0.5_)** | | | |
| **Time [s]** | | **Peak Counts** | **Peak Position** | **FWHM** |
| **Bare** | | 2642.46 | 419.09 | 7.16 |
| **0** | | 1052.98 | 419.01 | 7.59 |
| **60** | | 1451.45 | 419.03 | 7.69 |
| **240** | | 1501.72 | 418.99 | 7.50 |
| **3600** | | 1562.08 | 418.95 | 7.38 |
| **(c)** | **Raman Summary (Pd_0.75_Ag_0.25_)** | | | |
| **Time [s]** | | **Peak Counts** | **Peak Position** | **FWHM** |
| **Bare** | | 2748.39 | 419.01 | 7.56 |
| **0** | | 874.82 | 418.91 | 7.57 |
| **60** | | 1146.91 | 418.95 | 7.56 |
| **240** | | 1106.46 | 418.91 | 7.62 |
| **3600** | | 1327.79 | 418.85 | 7.49 |
